# Supplementary material for: The CENP-A chaperone complex spatially organizes centromeres
Source: bioRxiv. 2025 Nov 9:2025.11.07.687291. Preprint. [Version 1] doi: 10.1101/2025.11.07.687291 (PMC12637575; doi:10.1101/2025.11.07.687291)
Supplement: 1 [file NIHPP2025.11.07.687291V1-supplement-1.pdf]

bioRxiv preprint doi: https://doi.org/10.1101/2025.11.07.687291; this version posted November 9, 2025. The copyright holder for this preprint (which was not certified by peer review) is the author/funder, who has granted bioRxiv a license to display the preprint in perpetuity. It is made available under aCC-BY 4.0 International license.

Supplemental Figure 1

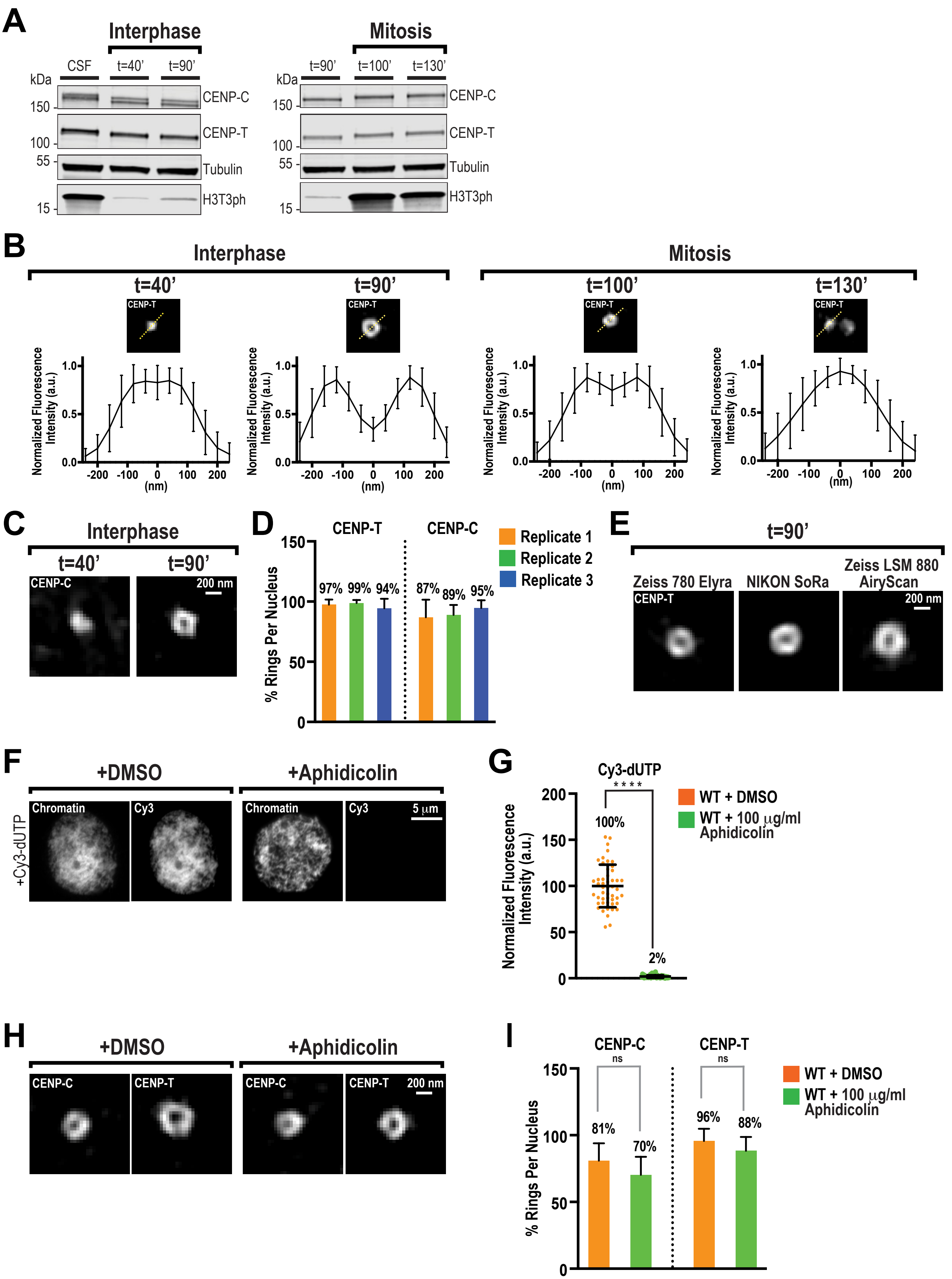

bioRxiv preprint doi: https://doi.org/10.1101/2025.11.07.687291; this version posted November 9, 2025. The copyright holder for this preprint (which was not certified by peer review) is the author/funder, who has granted bioRxiv a license to display the preprint in perpetuity. It is made available under aCC-BY 4.0 International license.

Supplemental Figure 2

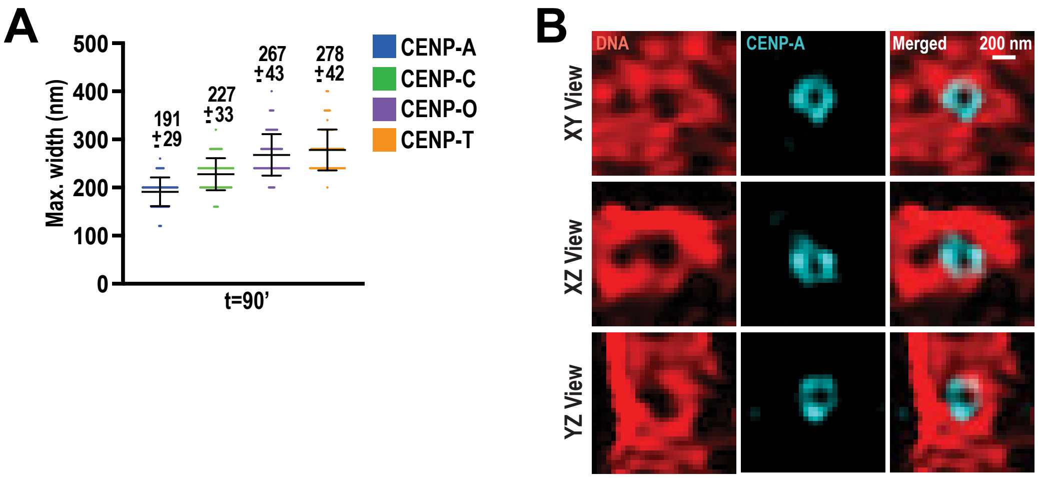

bioRxiv preprint doi: https://doi.org/10.1101/2025.11.07.687291; this version posted November 9, 2025. The copyright holder for this preprint (which was not certified by peer review) is the author/funder, who has granted bioRxiv a license to display the preprint in perpetuity. It is made available under a CC-BY 4.0 International license.

Supplemental Figure 3

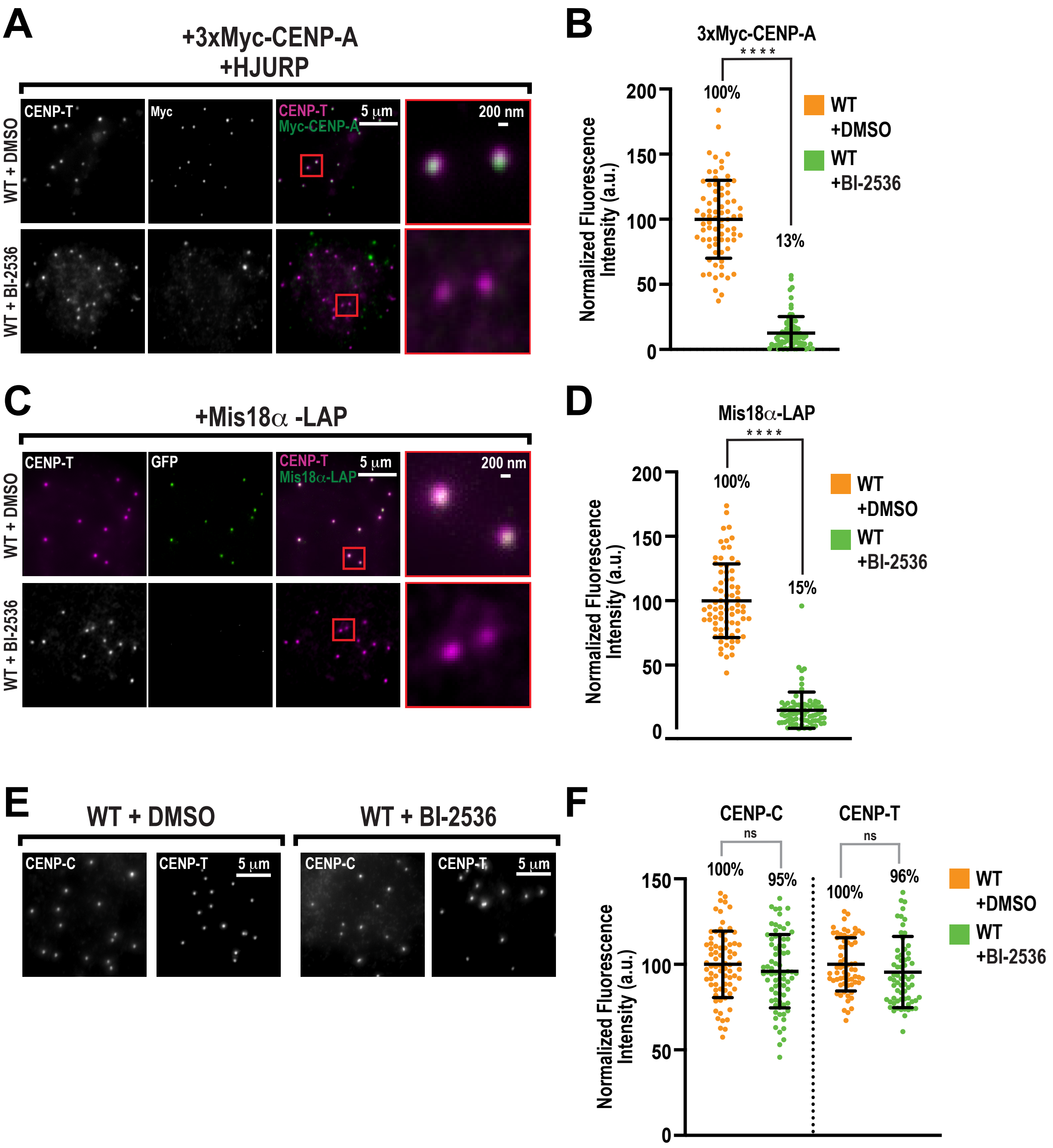

bioRxiv preprint doi: https://doi.org/10.1101/2025.11.07.687291; this version posted November 9, 2025. The copyright holder for this preprint (which was not certified by peer review) is the author/funder, who has granted bioRxiv a license to display the preprint in perpetuity. It is made available under a CC-BY 4.0 International license.

Supplemental Figure 4

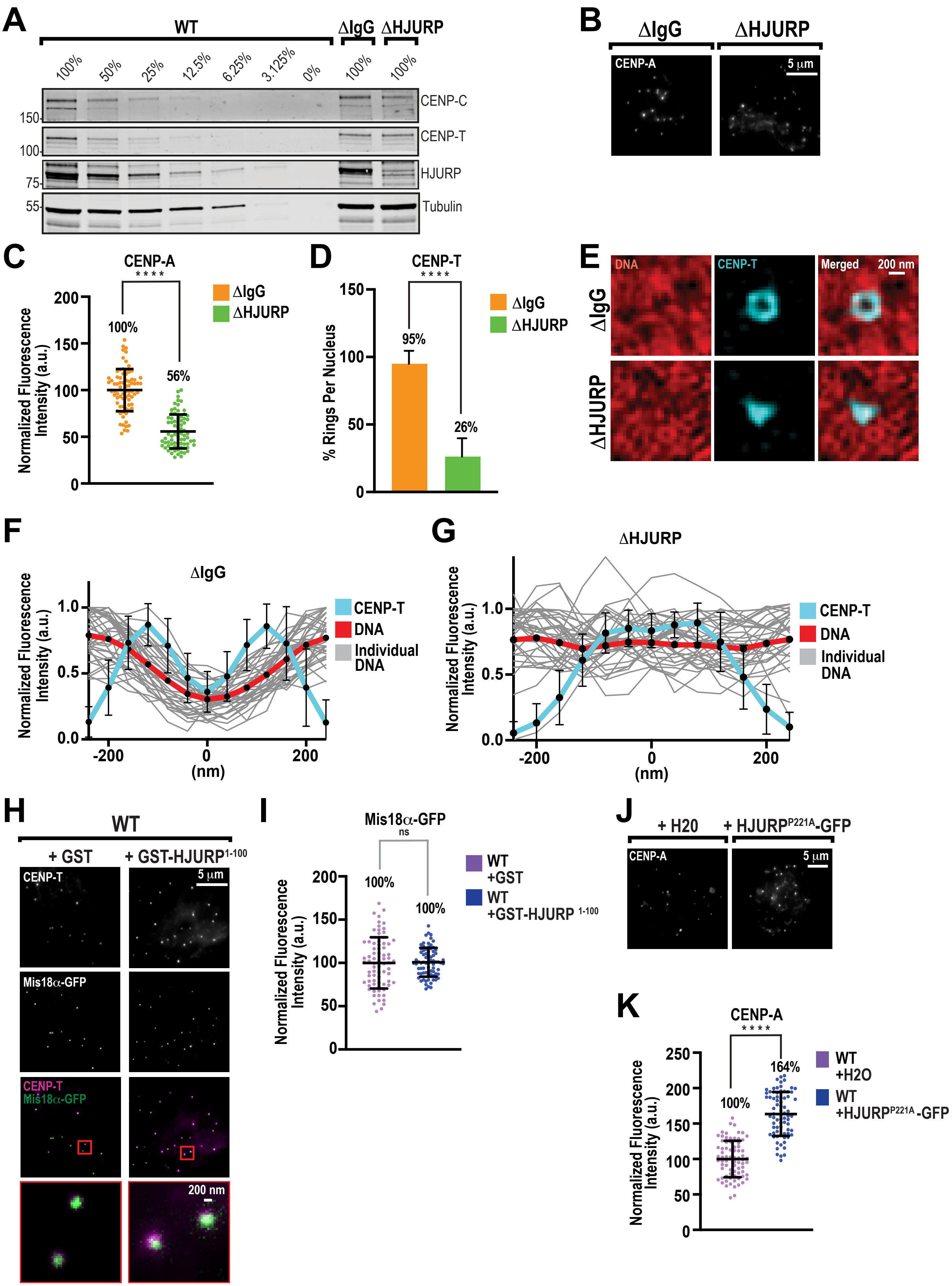

Supplemental Figure 5

bioRxiv preprint doi: https://doi.org/10.1101/2025.11.07.687291; this version posted November 9, 2025. The copyright holder for this preprint (which was not certified by peer review) is the author/funder, who has granted bioRxiv a license to display the preprint in perpetuity. It is made available under aCC-BY 4.0 International license.

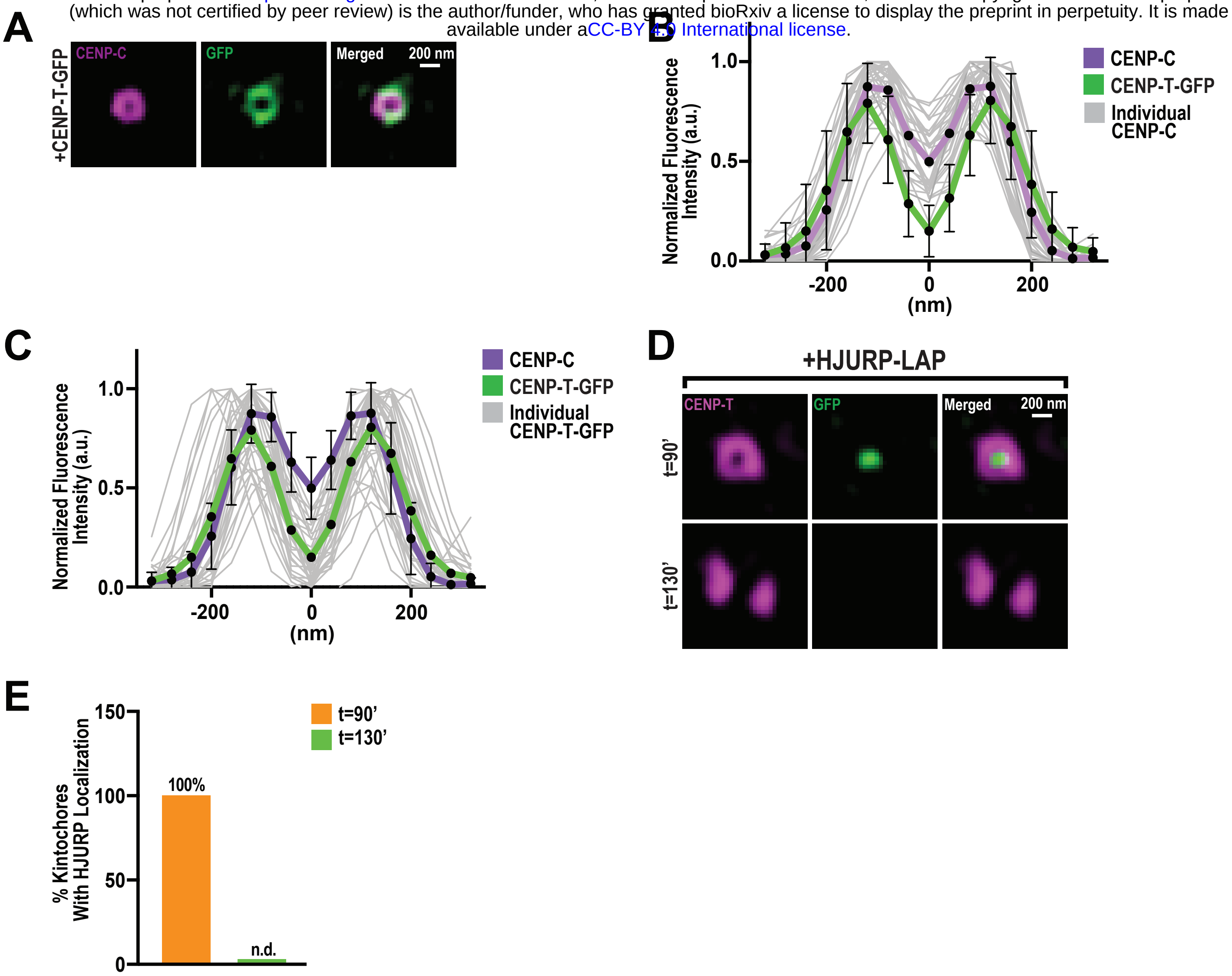

Supplemental Figure 6

bioRxiv preprint doi: https://doi.org/10.1101/2025.11.07.687291; this version posted November 9, 2025. The copyright holder for this preprint (which was not certified by peer review) is the author/funder, who has granted bioRxiv a license to display the preprint in perpetuity. It is made available under aCC-BY 4.0 International license.

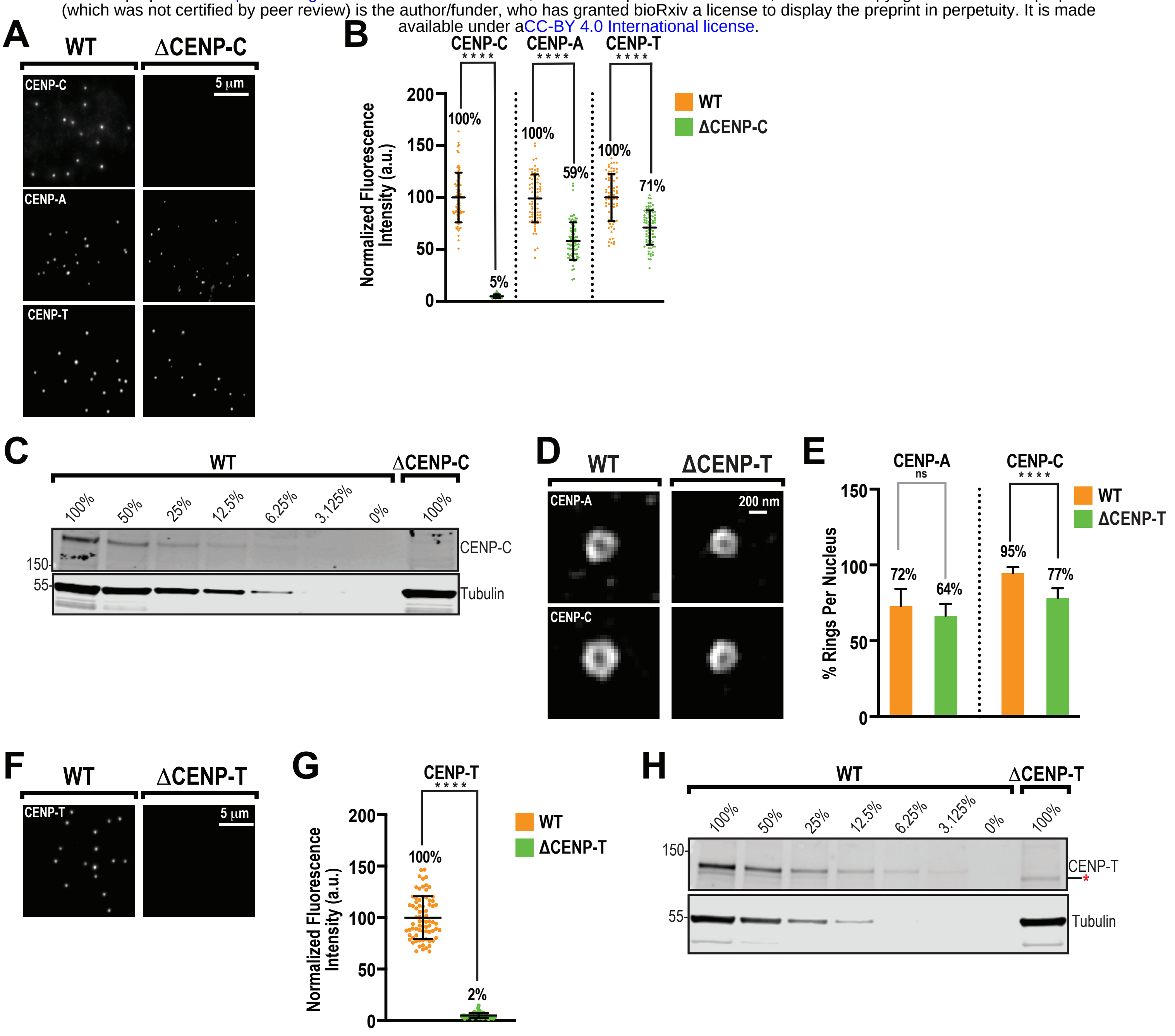

bioRxiv preprint doi: https://doi.org/10.1101/2025.11.07.687291; this version posted November 9, 2025. The copyright holder for this preprint (which was not certified by peer review) is the author/funder, who has granted bioRxiv a license to display the preprint in perpetuity. It is made available under aCC-BY 4.0 International license.

Supplementary Figure Legends

Fig. S1. Centromeric chromatin architecture changes during the cell cycle but is independent of DNA replication. (**A**) Western blot for CENP-C, CENP-T, Tubulin, and Histone H3 phosphorylation (H3T3ph) for samples shown in Fig. 1A. CSF (cytostatic factor) *Xenopus* egg extracts are arrested in M phase without prior cycling through interphase. (**B**) Averaged and aligned linescan quantification of CENP-T fluorescence at centromeres from Fig. 1A. Each individual trace (not shown) was aligned by its central minimum or maximum and normalized to the maximum intensity, and the average trace plotted on this scale. Insets illustrate the line scanning method. n=75 kinetochores for each condition. Error bars represent SD. (**C**) Representative 3D-SIM images of CENP-C on interphase nuclei at *t*=40 and 90' assembled from WT extract. Scale bar is 200 nm. (**D**) Percentage of observable ring-like structures per nucleus for CENP-C and CENP-T from three independent experiments. n=10 nuclei per condition, and n≥145 centromeres total. Error bars represent SD. (**E**) Representative super-resolution images of CENP-T on interphase nuclei at *t*=90, in WT extract acquired on the indicated microscope platforms. Scale bar is 200 nm. (**F**) Representative fluorescence images of chromatin (DAPI) and Cy3-dUTP on interphase nuclei, at *t*=90', assembled from extract treated with DMSO or Aphidicolin. Scale bar is 5 µm. (**G**) Quantification of fluorescence intensity of Cy3-dUTP fluorescence shown in (F), normalized to DMSO. n = 50 nuclei per condition. A.U., arbitrary units. Error bars represent SD, ****, $P < 0.0001$. (**H**) Representative 3D-SIM images of CENP-C and CENP-T on interphase nuclei, at *t*=90', assembled from extract treated with DMSO or Aphidicolin. Scale bar is 200 nm. (**I**) Quantification of percentage of CENP-C and CENP-T rings per nucleus following treatment with DMSO or Aphidicolin, shown in (H). n=10 nuclei per condition, and n≥145 centromeres total. Error bars represent SD. NS = Not significant

Fig. S2. 3D-SIM and deep learning analysis of CCAN and CENP-A structures.
(**A**) Average peak-to-peak distances of CENP-A, CENP-C, CENP-O, and CENP-T signals from linescans of 3D-SIM images of interphase nuclei at t=90', with representative images shown in Fig. 1B. n = 75 centromeres or kinetochores were quantified. Error bars represent SD. (**B**) Axial resolution enhancement of 3D-SIM images of CENP-A (cyan) and DNA (DAPI; red) from deep learning prediction. Representative XY, XZ, and YZ views of CENP-A and DNA are shown. Scale bar is 200 nm.

Fig. S3. Inhibition of Plk1 activity blocks new CENP-A assembly in *Xenopus* egg extracts.
(**A**) Representative immunofluorescence images of CENP-T (magenta) and 3XMyc-CENP-A (green) on interphase nuclei, at t=90', assembled from extracts containing 3X-Myc-CENP-A and HJURP mRNA and treated with DMSO or BI-2536. Higher magnification views of the red boxed regions are shown in the last column. Scale bar is 5 µm, except for higher magnification views (200 nm). (**B**) Quantification of fluorescence intensity of 3XMyc-CENP-A shown in (A) normalized to DMSO. n = 75 kinetochores per condition. A.U., arbitrary units. Error bars represent SD. (**C**) Representative immunofluorescence images of CENP-T (magenta) and Mis18α-GFP (green) on interphase nuclei, at t=90', assembled from extracts containing *in vitro* translated Mis18α-LAP treated with either DMSO or BI-2536. Higher magnification views of the red boxed regions are shown in the last column. Scale bar is 5 µm, except for higher magnification views (200 nm). (**D**) Quantification of fluorescence intensity of Mis18α-GFP shown in (C) normalized to DMSO. n = 75 kinetochores per condition. A.U., arbitrary units. Error bars represent SD. (**E**) Representative immunofluorescence images of CENP-C and CENP-

bioRxiv preprint doi: https://doi.org/10.1101/2025.11.07.687291; this version posted November 9, 2025. The copyright holder for this preprint (which was not certified by peer review) is the author/funder, who has granted bioRxiv a license to display the preprint in perpetuity. It is made available under aCC-BY 4.0 International license.

T on interphase nuclei, at *t*=90', assembled from extract treated with DMSO or BI-2536. Scale bar is 5 μm. (**F**) Quantification of fluorescence intensity of CENP-C and CENP-T shown in (E), normalized to DMSO. n=75 kinetochores per condition. A.U., arbitrary units. Error bars represent SD.

Fig. S4. HJURP, but not its role in CENP-A assembly, is required for centromere organization in interphase. (**A**) Western blot for CENP-C, CENP-T, HJURP, and Tubulin in WT, ΔIgG, and ΔHJURP extracts from the samples shown in Fig. 3A. (**B**) Representative immunofluorescence images of CENP-A on interphase nuclei, at *t*=90', assembled from ΔIgG and ΔHJURP extracts. Scale bar is 5 μm. (**C**) Quantification of fluorescence intensity of CENP-A shown in (B), normalized to ΔIgG. n=75 centromeres per condition. A.U., arbitrary units. Error bars represent SD, ****, $P < 0.0001$. (**D**) Percentage of observable CENP-T ring-like structures per nucleus for conditions in Fig. 3A. n=10 nuclei per condition, and n≥145 centromeres total. Error bars represent SD, ****, $P < 0.0001$. (**E**) Representative 3D-SIM images of CENP-T (cyan) and DNA (DAPI; red) on interphase nuclei, at *t*=90', assembled in ΔIgG or ΔHJURP extracts. Scale bar is 200 nm. **(F**) Linescan quantification of CENP-T and DNA fluorescence at kinetochores in ΔIgG condition from (E). Bold lines indicate average intensity, and thin gray lines indicate individual traces of DNA fluorescence. Each trace was normalized to the maximum intensity and aligned to the central minimum of CENP-T average intensity. n=35 kinetochores. Error bars represent standard deviation (SD). (**G**) Linescan quantification of CENP-T and DNA fluorescence at kinetochores in ΔHJURP condition from (E). Bold lines indicate average intensity, and thin gray lines indicate individual traces of DNA fluorescence. Each trace was normalized to the maximum intensity and aligned to the central maximum of CENP-T average intensity. n=35 kinetochores. Error bars represent standard deviation (SD). (**H**) Representative immunofluorescence images of CENP-T (magenta) and Mis18α-GFP (green) on interphase nuclei, at t=90', assembled from extracts containing *in vitro* translated Mis18α-GFP treated with GST or GST-HJURP$^{1\text{-}100}$. Higher magnification views of the red boxed regions are shown in the last column. Scale bar is 5 μm, except for higher magnification views (200 nm). (**I**) Quantification of fluorescence intensity of Mis18α-GFP shown in (H) normalized to GST. n= 75 kinetochores per condition. A.U., arbitrary units. Error bars represent SD. (**J**) Representative immunofluorescence images of CENP-A on interphase nuclei, at *t*=90', assembled from extracts containing water (H2O) or HJURP^{P221A}-GFP mRNA. Scale bar is 5 μm. (**K**) Quantification of fluorescence intensity of CENP-A shown in (J), normalized to H2O. n=75 centromeres per condition. A.U., arbitrary units. Error bars represent SD, ****, $P < 0.0001$.

Fig. S5. HJURP is evicted in mitosis, which correlates with the collapse of centromere organization. (**A**) Representative 3D-SIM images of CENP-C (magenta) co-stained with GFP (green) on interphase nuclei, at *t*=90', assembled from extracts containing *in vitro* translated CENP-T-GFP. Scale bar is 200 nm. (**B**) Line scan quantification of CENP-C and CENP-T-GFP fluorescence at centromeres from (A). Bold lines indicate average intensity, and thin gray lines indicate individual traces of CENP-C fluorescence. Each trace was aligned by the central minimum and normalized to the maximum intensity. n=75 kinetochores. Error bars represent SD. (**C**) Line scan quantification of CENP-C and CENP-T-GFP fluorescence at centromeres from (A). Bold lines indicate average intensity, and thin gray lines indicate individual traces of CENP-T-GFP fluorescence. Each trace was aligned by the central minimum and normalized to the maximum intensity. n=75 kinetochores. Error bars represent SD. (**D**) Representative 3D-SIM images of CENP-T (magenta) and HJURP-GFP (green) on interphase nuclei and mitotic

bioRxiv preprint doi: https://doi.org/10.1101/2025.11.07.687291; this version posted November 9, 2025. The copyright holder for this preprint (which was not certified by peer review) is the author/funder, who has granted bioRxiv a license to display the preprint in perpetuity. It is made available under aCC-BY 4.0 International license.

chromosomes at t = 90' (interphase) and 130'(metaphase), assembled from WT extract containing *in vitro* translated HJURP-GFP. (**E**) Percentage of centromeres with HJURP present on interphase nuclei and mitotic chromosomes shown in (D). n=75 kinetochores in each condition.

Fig. S6. CENP-C, but not CENP-T, is required for centromere architecture in interphase. (**A**) Representative immunofluorescence images of CENP-C, CENP-A, and CENP-T on interphase nuclei, at t=90', assembled from WT and CENP-C depleted (ΔCENP-C) extracts for the samples shown in Fig. 4D. Scale bar is 5 µm. (**B**) Quantification of fluorescence intensity of CENP-C, CENP-A, and CENP-T shown in (A) normalized to WT. n = 75 centromeres or kinetochores per condition. A.U., arbitrary units. Error bars represent SD, ****, $P < 0.0001$. (**C**) Western blot for CENP-C and Tubulin in WT and ΔCENP-C extracts from the samples shown in (A) and Fig. 4D. (**D**) Representative 3D-SIM images of CENP-A and CENP-C on interphase nuclei, at t=90', assembled from WT and CENP-T depleted (ΔCENP-T) extracts. Scale bar is 200 nm. (**E**) Percentage of observable CENP-A or CENP-C ring-like structures per nucleus for conditions in (D). n=8 nuclei per condition, and n≥129 centromeres total. Error bars represent SD, ****, $P < 0.0001$. (**F**) Representative immunofluorescence images of CENP-T on interphase nuclei, at t=90', assembled from WT and ΔCENP-T extracts. Scale bar is 5 µm. (**G**) Quantification of fluorescence intensity of CENP-T immunofluorescence from (F), normalized to WT. n = 75 kinetochores per condition. A.U., arbitrary units. Error bars represent SD, ****, $P < 0.0001$. (**H**) Western blot for CENP-T and Tubulin in WT and ΔCENP-T extracts from the samples shown in (D). Asterisk indicates a non-specific band.
